# Ovarian endometriomas and IVF: a retrospective case-control study

**DOI:** 10.1186/1477-7827-9-81

**Published:** 2011-06-17

**Authors:** Francesca Bongioanni, Alberto Revelli, Gianluca Gennarelli, Daniela Guidetti, Luisa Delle Delle Piane, Jan Holte

**Affiliations:** 1 LIVET Infertility and IVF Clinic, Torino, Italy; 2 Reproductive Medicine and IVF Unit, Department of Obstetrical and Gynecological Sciences, University of Torino, OIRM-S, Anna Hospital, Torino, Italy; 3 Carl von Linne' Clinic, Uppsala, Sweden

## Abstract

We performed this retrospective case-control study analyzing 428 first-attempt in vitro fertilization (IVF) cycles, among which 254 involved women with a previous or present diagnosis of ovarian endometriosis. First, the results of these 254 cycles were compared with 174 cycles involving patients with proven non-endometriotic tubal infertility having similar age and body mass index. Women with ovarian endometriosis had a significantly higher cancellation rate, but similar pregnancy, implantation and delivery rates as patients with tubal infertility. Second, among the women with ovarian endometriosis, the women with a history of laparoscopic surgery for ovarian endometriomas prior to IVF and no visual endometriosis at ovum pick-up (n = 112) were compared with the non-operated women and visual endometriomas at ovum pick-up (n = 142). Patients who underwent ovarian surgery before IVF had significantly shorter period, lower antral follicle count and required higher gonadotropin doses than patients with non-operated endometriomas. The two groups of women with a previous or present ovarian endometriosis did, however, have similar pregnancy, implantation and live birth rates. In conclusion, ovarian endometriosis does not reduce IVF outcome compared with tubal factor. Furthermore, laparoscopic removal of endometriomas does not improve IVF results, but may cause a decrease of ovarian responsiveness to gonadotropins.

## Background

Laparoscopic stripping of endometriomas before IVF/ICSI treatment in order to improve its outcome is widespread in everyday clinical practice. This procedure is, however, not based on clinical evidence[1-3]. Although a previous metanalysis [4], showed reduced pregnancy rates after IVF in women with ovarian endometriosis compared to patients undergoing IVF for other indications [5], other later studies could not confirm this finding [6-9]. When the influence of the surgical removal of endometriomas on IVF outcome was studied, operated patients obtained IVF results comparable to women without previous surgical intervention [10-15]. Furthermore, some results suggest that the surgical intervention may have a negative effect on ovarian reserve [12,16-18] and hence putatively compromise treatment outcome and long-term fertility.

Thus, the possible impact of ovarian endometriosis on ART results remain a controversial issue. The aim of the present study was to retrospectively analyze a large number of IVF/ICSI cycles and to evaluate the treatment outcome (a) in patients with a diagnosis of ovarian endometriosis in comparison to that in patients with tubal infertility and (b) in women with a history of laparoscopic removal of ovarian endometriomas prior to IVF compared with the outcome in women without previous surgical intervention and a visual endometrioma at OPU.

## Methods

### Patients

A total number of 8623 first-attempt IVF cycles performed in three IVF units were retrospectively analyzed. In total, 254 cycles were found to involve women diagnosed with ovarian endometriosis. Of these, 142 women had never undergone any ovarian surgery and displayed one or more in situ ovarian endometriomas of small to medium size (< = 6 cm in diameter; Group A), and 112 women underwent IVF after the laparoscopic removal of one or more endometrioma(s) of comparable size (< = 6 cm in diameter) by the classical "stripping" technique (Group B). These women did not have visual endometriomas at the time of OPU. The proportion of patients with bilateral endometriomas was 14.1% in Group A (20 out of 142) and 19.6% in group B (22 out of 112), a difference not statistically significant. In case of bilateral or multiple endometriomas, their diameter was summed: in all cases, the total diameter did not exceed 6 cm. Endometriosis patients in Group B were operated because they were symptomatic, whereas those in Group A were not operated before IVF because they were no or less symptomatic, although they had endometriomas of similar size.

In all patients submitted to laparoscopic ovarian resection, the diagnosis of ovarian endometriosis was histologically confirmed. In women who had not been operated before IVF (Group A), the diagnosis of ovarian endometriosis was based on transvaginal ultrasound (evidence of an adnexal mass with diffuse low level echoes without neoplastic or acute hemorrhage features; [19]) and the AFS stage was estimated to be II-III in all cases. None of the patients operated for ovarian endometriosis received GnRH-agonists or other medical treatments prior to or after operation.

The control group consisted of 174 women who underwent IVF treatment during the same time period, with laparoscopically diagnosed tubal factor and without any evidence of ovarian endometriosis. These women covered similar ranges of age and BMI as the endometriosis patients (Group C).

### IVF procedure

Ovarian stimulations were conducted with daily subcutaneous injections of individual starting doses of rFSH (Follitropin alfa (Merck-Serono, Geneva, Switzerland) or Follitropin beta (Organon, Oss, the Netherlands) or hMG (Meropur/Menopur, Ferring, Switzerland) at appropriate doses (100-450 IU), estimated according to the woman'sage, the antral follicle count and the basal (day 3) FSH. The long GnRH-agonist down-regulation protocol was used (Nafarelin-Pfizer Inc., New York, USA) 400 mg nasally twice daily, or buserelin (Sanofi-Aventis, Paris, France) 0.3 mg nasally four times daily); in both cases half the dose was administered during ovarian stimulation. Ovarian response to gonadotropins was monitored by transvaginal ultrasound plus serum E2 measurement every third day from stimulation day 7. Ovulation was triggered by injecting 10,000 IU hCG s.c. when the leading follicle reached 18 mm, with appropriate serum E2 levels. Transvaginal ultrasound-guided oocyte aspiration (OPU) was performed approximately 36 hours after hCG injection under local anaesthesia (paracervical block). Either IVF or ICSI was performed according tothe clinical indication. After cultivation, embryos were transferred on Day 2 or 3 after ovum pick-up (OPU). Luteal phase support was given vaginally to all patients for 2 weeks from embryo transfer (progesterone vagitories (Apoteket AB, Stockholm, Sweden) 1200 mg or gel (Merck-Serono, Geneva, Switzerland) 180 mg daily). Pregnancy was defined as the visualization of a gestational sac at vaginal ultrasound investigation in gestational week 7. All data were de-identified ahead of analysis. The study did not in any way alter our routine IVF/ICSI protocols, nor did it involve any additional intervention at treatment. All data were prospectively collected with the intention to evaluate impact on treatment outcome.

### Ovarian surgery technique

A four-port laparoscopy technique was used: an 11 mm trocar was inserted through a short umbilical incision and connected to a video monitor (WideVieuw™HD Karl Storz Endoscope); two additional lateral 5 mm operating ports and a central sovrapubic 5-10 mm operating port were also inserted. The pneumo-peritoneum was achieved by inflating CO2 (10 mmHg).

To excide endometriomas, an incision was performed at the antimesenteric site of the affected ovary using bipolar cautery; then, the endometrioma was drained with aspiration and the pseudo-capsule was dissected by gentle traction and countertraction using two 5 mm grasping forceps ("stripping"). The bleeding at the stripping site was stopped by bipolar cautery, only when necessary and very carefully in order to avoid unnecessary thermal damage to the healthy ovarian tissue.

### Assessment of ovarian sensitivity to FSH

To assess ovarian sensitivity to FSH, the ratio between the number of retrieved oocytes and the number of FSH IU (x100) was calculated. This variable was defined "ovarian sensitivity", as it shows the effective ovarian response to FSH stimulation, independently on the total amount of administered FSH. Patients with more abundant ovarian follicular reserve tend to display higher ovarian sensitivity to exogenous FSH, whereas women with a low ovarian reserve usually have a lower ovarian sensitivity.

### Statistical analysis

Data are expressed as the mean ± SEM or as percentages when required. Statistical comparisons among groups were performed using the Fisher exact test, Yeats' corrected χ^2^, Wilcoxon's test or Student's t test, as appropriate. The JMP software was used for statistical elaboration. Significance was defined as a p value < 0.05.

## Results

### IVF outcome in patients with ovarian endometriosis vs. patients with tubal factor

The basal characteristics of patients with ovarian endometriosis (Group A), previously operated of endometrioma excision (Group B) and tubal infertility (Group C, controls) are shown in Table 1.

**Table 1:** Clinical characteristics of patients having one or more in situ endometrioma(s) at the time of IVF (Group A) vs. those previously operated for laparoscopic endometrioma(s) removal (Group B) vs. women with tubal infertility (Group C, controls)

	Group A	Group B	Group C	p
*Patients*	142	112	174	
*Age (yrs)*	33.8 ± 3.1	33.6 ± 4.4	34.0 ± 3.1	ns
*BMI (kg/m^2^)*	22.7 ± 3.2	22.4 ± 3.2	23.1 ± 3.3	ns
*Smoke (%)*	11.8	16.1	15.3	ns
*Mean period (days)*	28.8 ± 4.0	27.2 ± 4.1	28.5 ± 3.1	< 0.005^1^ < 0.004^2^
*Infertility duration (years)*	4.0 ± 2.5	3.9 ± 2.9	3.6 ± 0.3	ns
*Associated male factor (%)*	13.8	19.1	13.7	ns
*Antral follicle count*	16.9 ± 11.1	11.7 ± 9.4	16.6 ± 9.5	< 0.001^1,2^
*FSH day 3 level (U/l)*	7.2 ± 3.9	7.9 ± 4.2	6.6 ± 3.5	ns

^1^Group A vs Group B, ^2^Group B vs Group C, ns: not significant.

The groups were comparable for age, BMI, infertility duration, smoking habits, mean period, prevalence of associated male factor, and basal (day 3) FSH levels. The basal antral follicle count was significantly lower in women previously submitted to the excision of ovarian endometriosis (Group B) than in women with ovarian endometrioma(s) (Group A) or tubal factor infertility (Group C).

The outcome of IVF in patients affected by ovarian endometriosis versus woman with tubal factor is shown in Table 2. Women with ovarian endometriosis showed a significantly higher cancellation rate, but those who completed ovarian stimulation had a similar yield of oocytes at OPU and a comparable ovarian sensitivity compared to control subjects. Fertilization rates were similar, but women with endometriosis showed a lower percentage of cycles with complete failure of fertilisation. Overall, the implantation, pregnancy and live-birth rates per started cycle, OPU, and ET were similar in the groups.

**Table 2:** IVF outcome of patients having one or more in situ endometrioma(s) at the time of IVF (Group A) vs. those previously operated for laparoscopic endometrioma(s) removal (Group B) vs. woman with tubal infertility (Group C, controls)

	Group A	Group B	Group C	p
*Cancellation rate (%)*	7.5	9.8	2.9	< 0.02^2,3^
*Total FSH dose (IU)*	2339 ± 1248	3298 ± 1404	2537 ± 1090	< 0.001^1,3^
*N. of retrieved oocytes*	9.4 ± 4.3	8.2 ± 5.3	9.6 ± 4.0	< 0.03^3^
*MII oocytes (%)*	71.2	68.8	66.9	ns
*Ovarian sensitivity*	5.6 ± 3.2	3.5 ± 3.5	5.0 ± 3.0	< 0.001^1,3^
*Type of treatment (%)*				
*IVF*	77	72	68	ns
*ICSI*	19	23	25	ns
*Combined*	4	5	7	ns
*Fertilization rate (%)*	67.7	73.4	70.2	ns
*Cycles with no fertilization (%)*	6.6	8.2	10.0	< 0.04^2^
*Number of embryos transferred*	2.0 ± 0.5	2.1 ± 0.6	2.2 ± 0.4	ns
*Pregnancy rate/ started cycle (%)*	41.5	36.6	35.0	ns
*Pregnancy rate/OPU (%)*	45.0	40.6	36.1	ns
*Pregnancy rate/ ET (%)*	48.4	44.1	40.1	ns
*Implantation rate (%)*	24.2	24.6	22.1	ns
*Live-birth rate/ET (%)*	34.6	25.8	30.8	ns

^1^Group A vs Group B, ^2^Group B vs Group C, ^3^Group A vs Group C, ns: not significant.

### IVF outcome in patients with in situ ovarian endometrioma(s) vs. patients previously operated for ovarian endometrioma(s)

The clinical characteristics of patients having one or more in situ ovarian endometriomas at the time of IVF (Group A) and of women who had been previously operated for endometrioma removal (Group B) are shown in Table 1. Operated women had a shorter mean period and lower antral follicle counts than women with in situ endometrioma(s).

Women with previous ovarian surgery required higher total FSH doses than patients without previous surgery, but had comparable numbers of oocytes at OPU (Table 2). Thus, ovarian sensitivity was lower in operated women. The implantation rate, pregnancy rate and live-birth rate per started cycle, OPU, and ET were similar in the two groups.

## Discussion

The present study retrospectively analyzed a large cohort of patients undergoing IVF in the years 2004-2009, identifying 254 patients with a previous or present diagnosis of ovarian endometriosis. With the limitations of a retrospective study (although on a remarkably large number of observations), our results suggest that previous or present ovarian endometriosis does not impair success rates at IVF/ICSI, and that ovarian surgery for endometriosis does not result in improved ART outcome, but, on the contrary, may compromise ovarian reserve.

Laparoscopic stripping of ovarian endometriomas as an intervention to improve fertility is a widespread clinical practice, not only to improve natural fertility, but also to improve IVF outcome. This surgical strategy is used because of the following reasons: a) older studies suggested that patients with ovarian endometriosis had poorer IVF outcome than women with other infertility causes; b) some data suggest that spontaneous fecundity may improve after laparoscopic cystectomy [1]; c) some argue that puncturing an endometrioma during oocyte retrieval could spread endometriotic cells in the abdominal cavity or cause a pelvic infection.

The risk of complications linked to the puncture of ovarian endometriomas is, however, minimal: infections have been reported only sporadically [20,21], and indeed a study in which ovarian endometriomas were intentionally punctured and aspirated at the time of oocyte retrieval reported no complications [22]. Furthermore, aspiration of endometriomas followed by local injection of methotrexate [23] or alcoholic solutions [24,25] is considered a therapeutic option for ovarian endometriosis.

As for IVF outcome in women with a diagnosis of ovarian endometriosis, the published data exhibit varying results. A meta-analysis from 2002 including 22 studies showed a reduced pregnancy rate after IVF in women with endometriosis compared to treatments in women with other infertility causes, and also showed a linear (inverse) relationship between the stage of the disease and the pregnancy rate [4]. However, several subsequent studies, including a large epidemiological survey [9], reported similar IVF outcome in patients with ovarian endometriosis as in women with other infertility causes [6,8,26,27]. A recent study comparing patients with endometriomas with women with non-endometriotic ovarian cysts suggested that ovarian endometriosis was associated with poorer embryo quality, although the pregnancy rate was unaffected [28]. Intervention studies investigating the effectiveness of laparoscopic removal of ovarian endometriosis as a tool to improve subsequent IVF results also show mainly negative results. Several reports showed that the outcome of IVF in patients previously submitted to laparoscopic stripping of endometriomas was similar to that of endometriosis-free controls [27,29-32]. As these studies did not include a group of patients with endometriomas who had not been subject to surgery, the impact of operating ovarian endometriosis *per se *could not be evaluated. In line with our results, a recent metanalysis showed that the outcome of IVF was similar in patients with in situ ovarian endometriomas as in endometriosis-free women [7]. In 30 infertile patients that served as their own controls as they were treated with IVF both before and after surgical treatment of ovarian endometriosis, Shahine [15] showed that embryo quality on day 3 was not improved after ovarian surgery, and IVF results remained comparable to those obtained before the operation. A recent meta-analysis including five studies comparing surgery vs. no treatment of endometrioma before IVF showed that there was no significant difference in the clinical pregnancy rate between the operated and the non-operated patients [13]. Only the study of Barri [33] reported a better IVF outcome in patients with ovarian endometriosis previously submitted to ovarian surgery vs. patients undergoing IVF as the first therapeutic option. Thus, most available data clearly show that surgical management of endometriomas gives no advantage for a subsequent IVF.

Our results showed that women operated for ovarian endometriosis exhibited several markers of a reduced ovarian reserve. Thus, cancellation rates, mean period [34], antral follicle counts and ovarian sensitivity to FSH/hMG were all reduced in the group of operated women. These findings are well in line with previous data. Patients previously submitted to laparoscopic cystectomy required a higher gonadotropin dose to achieve a similar ovarian response [12,16-18] or showed a lower oocyte yield [12,14,32,35-37]. In women operated for a monolateral ovarian endometrioma, it was reported that the operated ovary produced a lower number of follicles than the contralateral [38,39]. Indeed a histologically proven loss of functional ovarian tissue close to the cyst was well documented [40]. Moreover, Tinkanen [10] reported that non-operated patients had significantly more embryos and higher pregnancy and live birth rates than operated women. In a prospective, randomized trial, Demirol [12] showed that operated women required a higher FSH dose and a longer stimulation, obtained less oocytes and finally had similar IVF outcome as women with in situ endometriomas. Somigliana [14] reported that women operated for bilateral endometriotic ovarian cysts and subsequently submitted to IVF had a higher withdrawal rate for poor response, retrieved less oocytes despite the use of higher doses of gonadotropins, and had significantly lower pregnancy and delivery rates than non-endometriotic, never operated on the ovary, controls.

In conclusion, with the limitations of a retrospective study we show herein that the presence of ovarian endometriosis is not a cause of poorer IVF outcome and that laparoscopic stripping before IVF does not improve outcome. On the contrary, the operation may reduce ovarian reserve and increase the need for exogenous hormones to retrieve an adequate number of oocytes, thus increasing the overall cost of the treatment. Our observations, in line with most recent data, add evidence against laparoscopic ovarian surgery for endometriomas in asymptomatic patients who are candidates for IVF.

## Competing interests

The authors declare that they have no competing interests.

## Authors' contributions

FB, DG and LDP collected the data and provided the first draft of the manuscript. GG participated in the design of the study and performed the statistical analysis. AR and JH conceived of the study, participated in its design and coordination, and helped to draft the manuscript. All authors read and approved the final manuscript.
